# Development of a novel experimental technique for the measurement of residual wall layer thickness in water-oil displacement flows

**DOI:** 10.1038/s41598-023-31776-5

**Published:** 2023-03-20

**Authors:** Yao Zhang, Benjamin Barrouillet, Sachin M. Chavan, Hans Joakim Skadsem

**Affiliations:** 1 Department Energy and Petroleum Engineering, University of Stavanger, 4068 Stavanger, Norway; 2 Department Chemistry, Bioscience and Environmental Engineering, University of Stavanger, 4068 Stavanger, Norway

**Keywords:** Fluid dynamics, Applied physics, Techniques and instrumentation, Chemical engineering

## Abstract

The effective removal and displacement of fluids is important in many industrial and environmental applications, such as for operation and cleaning of process equipment, fluid injection in porous media for oil recovery or aquifer remediation, or for achieving subsurface zonal isolation in new or abandoned wells. The accurate measurement of the residual fluid wall film left behind after displacement by a cleaning fluid is a long-standing challenge, particularly so for very thin fluid films where the thickness can be of the order of micrometer. We focus on the characterization of oil films left on the wall of a horizontal pipe after the pipe has been displaced by water, and develop a novel, non-intrusive analytical technique that allows the use of relevant pipe materials. The oil that originally occupies the pipe is stained by a hydrophobic dye Nile red, and an intermediate organic solvent is used to collect the residual oil volume that remains after displacing the pipe with a known volume of water. Finally, ultraviolet-visible spectroscopy is used to measure the Nile red concentration in the collected fluid, which is proportional to the residual volume of oil in the pipe. We demonstrate the methodology by conducting experiments where the displacing fluid is injected at two different imposed velocities, and where the injected fluid volume is varied. As expected, we find a gradual thinning of the oil film with increasing injected fluid volume. We compare the measured film thicknesses to a displacement model based on the steady velocity profile in a pipe, and find that experiments consistently produce smaller film thicknesses. This developed technique allows quantification of displacement and cleaning mechanisms involved in immiscible displacements at laminar, transitional and turbulent regimes, for different non-Newtonian fluid pairs, and for different realistic pipe materials and surface roughnesses.

## Introduction

Two-phase and fluid displacement flows involving immiscible fluids are widespread in both industrial and biological applications^1^, such as the simultaneous flow of oil and gas in pipelines^2^, the displacement of oil from porous reservoir rocks by water injection^3,4^, or the flow of air through lung airways lined with a viscous fluid^5,6^. Depending on the application and the flowing conditions, immiscible two-phase flows can exhibit different fluid arrangements, with stratified flow, core-annular flow or bubble flow being relevant examples encountered in pipelines^2^. Our focus here is on immiscible liquid displacement from pipes, where water is injected to displace a resident oil that initially occupies the pipe. The study is in part motivated by similar displacement processes involved in the enterprise of constructing wells for petroleum production or geological sequestriation of carbon dioxide. Such wells require the placement of competent cementitious barriers for zonal isolation^7^. The barrier quality is linked to the complete displacement of the resident drilling fluid in the well, and to the effective hydraulic cleaning of solid bounding surfaces along the length of the barrier^8,9^.

Studies of fluid displacement linked to cementing and barrier placement have mainly focused on pairs of miscible fluids, and considered effects of geometry (inclination, annular clearance and eccentricity), placement conditions (flow rate, injection volume) and fluid properties (density and viscosity contrasts) on the evolution of the fluid-fluid interface and the displacement efficiency^7^. Recently, displacements involving *immiscible* liquids (water and oil) have been studied experimentally for inclined pipes^10,11^ and theoretically for inclined ducts^12^, revealing new instabilities and flow patterns compared to miscible displacements^10^. Most of the current research into displacement studies have been based around visual observation of fluid-fluid interfaces during displacement in transparent pipes and annuli. As such, the study of removal of thin wall films has largely been limited by the optical resolution of the instrumentation and by the refraction of light through curved wall surfaces. In this work, we present a novel and simple analytical method for estimating the residual oil volume left behind in a pipe after injecting a known volume of displacing water. The method allows measuring residual oil volumes that correspond to equivalent micrometer-sized film thicknesses using non-intrusive methods. The method is based on staining the oil-phase with a hydrophobic dye, and to use an organic solvent to collect the remaining oil after displacement of the pipe by injecting a known volume of water. We use Nile red as the hydrophobic dye and tetrahydrofuran (THF) as organic solvent. The hydrophobic property of Nile red has previously been utilized for selective staining and detection of lipid droplets^13^, for protein characterization^14^, and to measure the effectiveness of injectable aqueous filters for subsurface separation of non-aqueous phase liquids^15^. Finally, ultraviolet-visible (UV–Vis) spetrophotometry is subsequently used to measure the dye concentration in the THF solution, which in turn is proportional to the residual oil volume.

Various physical principles have in the past been used to measure the thickness of liquid films, including the liquid speed of sound (ultrasound measurement), liquid conductivity or radiation attenuation (gamma ray, neutron or X-ray measurement)^16^. More recently, Nile red was used as a stain or tracer in combination with fluorescence measurements to study the oil film removal due to wall shear in wall-driven (Couette) flow^17,18^. It was shown that the fluorescence measurement was capable of detecting dye concentrations corresponding to micrometer-sized wall films, and the experimental results highlighted the impact of wettability, metallurgy and surface roughness on wall film removal^17,18^. In this work, we apply similar techniques but focus on pressure-driven (Poiseuille) flows and utilize UV–Vis spectroscopy to measure the residual oil volume; UV–Vis spectrophotometer is easy to operate and widely available with high measurement accuracy. Even small UV–Vis spectrometers can give extremely accurate readings, which is crucial for research that deals with large amount of chemical solutions. We demonstrate the measurement technique using a mineral oil and water as the fluid pair, and perform displacement experiments at flowing conditions in the upper laminar regime. The methodology can be applied directly for future studies of how fluid displacement and wall cleaning is affected by non-Newtonian fluid viscosity, a turbulent flow regime or different pipe wall roughnesses.

## Methodology

### Experimental setup

**Figure 1:**
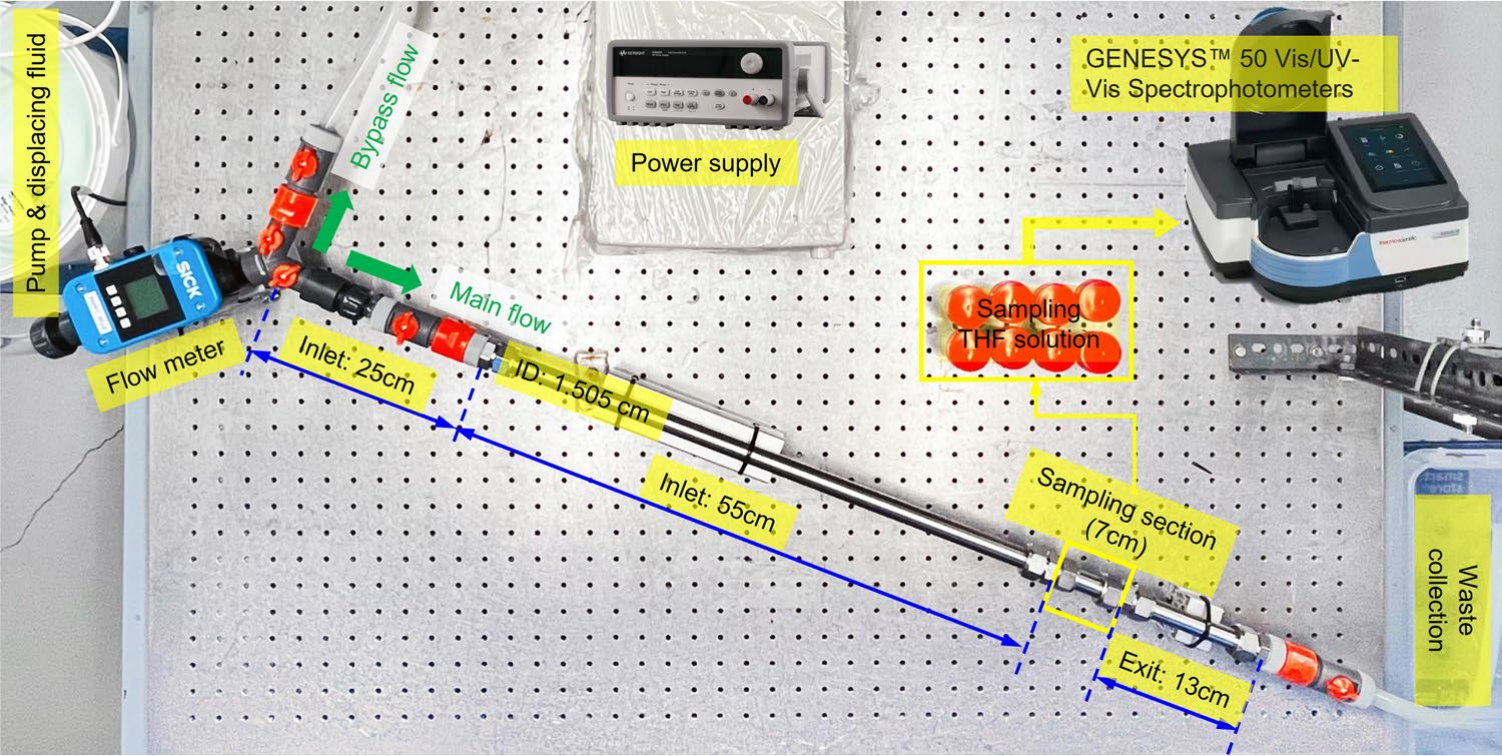
Layout of the experimental setup.

The experimental setup used to perform displacement experiments is shown in Fig. 1. The setup consists of three joined pipe segments that total a length of 1 m: (i) a 80 cm inlet section, (ii) a 7 cm sampling section, and (iii) a 13 cm exit section. The pipe segments are made of stainless steel and have an inner diameter of 15.05 mm. The joined segments make up a straight pipe that is arranged horizontally. Quick connectors at either side of the sampling section allow the user to easily retrieve this section after injection of the displacing fluid, with the residual oil film attached to the walls. A centrifugal pump controlled by an external power supply delivers the displacing fluid to the pipe, and the corresponding flow rate is measured using an ultrasonic flow meter, SICK Sensor Intelligence (Model FFUS15-1G1IO).

We perform displacement experiments where a mineral oil is displaced by the injection of a known volume of water at a fixed volumetric flow rate. To effectively measure the residual oil volume at the end of displacement, we stain the oil with a known concentration of Nile red, which is a hydrophobic dye soluble in oil and that can be easily identified through spectrometry. In addition, Nile red does not interact with other constituent and additives within the oil^19^. Nile red was therefore considered to be a suitable dye for immiscible displacement experiments. The Nile red dye (9-(Diethylamino)-5H-benzo[a]phenoxazin-5-one) in this study was aquired from TCI Europe.

**Figure 2:**
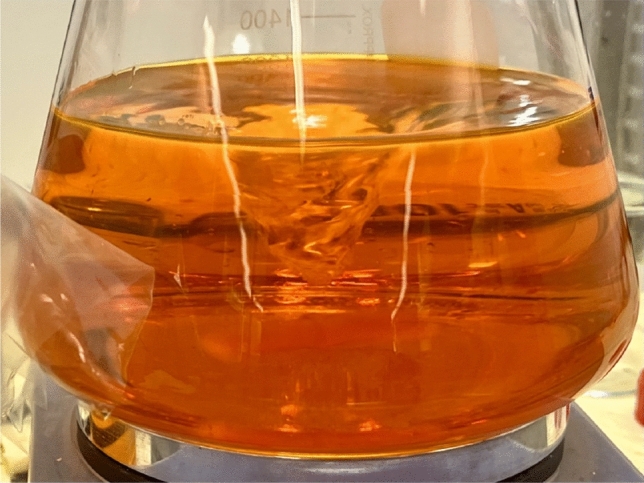
Sample Sipdril 4/0 stained with Nile red on a magnetic hot plate stirrer.

Since the volume of recovered oil after displacement was expected to be small, and typically corresponded to equivalent wall film thicknesses of the order of tens of micrometers, ensuring a homogeneous distribution of Nile red dye in the oil is critical to the accuracy of measurements. To ensure that the stained oil was a homogeneous mixture, an ultrasonic homogenizer (Model: Branson 450 Digital Sonifier) was used to mix Nile red in the mineral oil. The mixing process lasted for 15 min, until the color was considered even and uniformly distributed throughout the liquid. The well-mixed oil appeared transparent, and with a dark orange color, as shown in Fig. 2. After the oil was mixed with Nile red dye, it was kept on a magnetic hot plate stirrer operating at 600 revolutions per minute at a temperature of 25$^{\circ }$C.

After each displacement experiment, the test section containing residual oil stained with Nile red dye was submerged in the water-miscible organic solvent tetrahydrofuran (THF; > 99% stabilized, supplied by VWR Chemicals). The residual oil film was thus dissolved into the THF, and created a new solution containing a diluted concentration of Nile red dye. The concentration of Nile red in this solution was measured using UV–Vis spectroscopy (GENESYS 50 UV–Vis spectrophotometer by ThermoFisher Scientific) and a concentration calibration curve. The calibration curve and the conversion to an equivalent wall layer thickness are described next.

### Nile red concentration measurement

A calibration curve of absorption intensity as function of Nile red concentration is needed to record the concentration of recovered Nile red dye in the THF solution. To construct the calibration curves, 10 different known concentrations of Nile red in THF were prepared, and the absorption intensity measured as a function of wavelength for each of the prepared solutions, along with the pure THF solution. The wavelength corresponding to the maximum absorbance of the prepared solutions was chosen for analysis. This choice ensures accuracy and maximum sensitivity from UV–Vis measurements.

**Figure 3:**
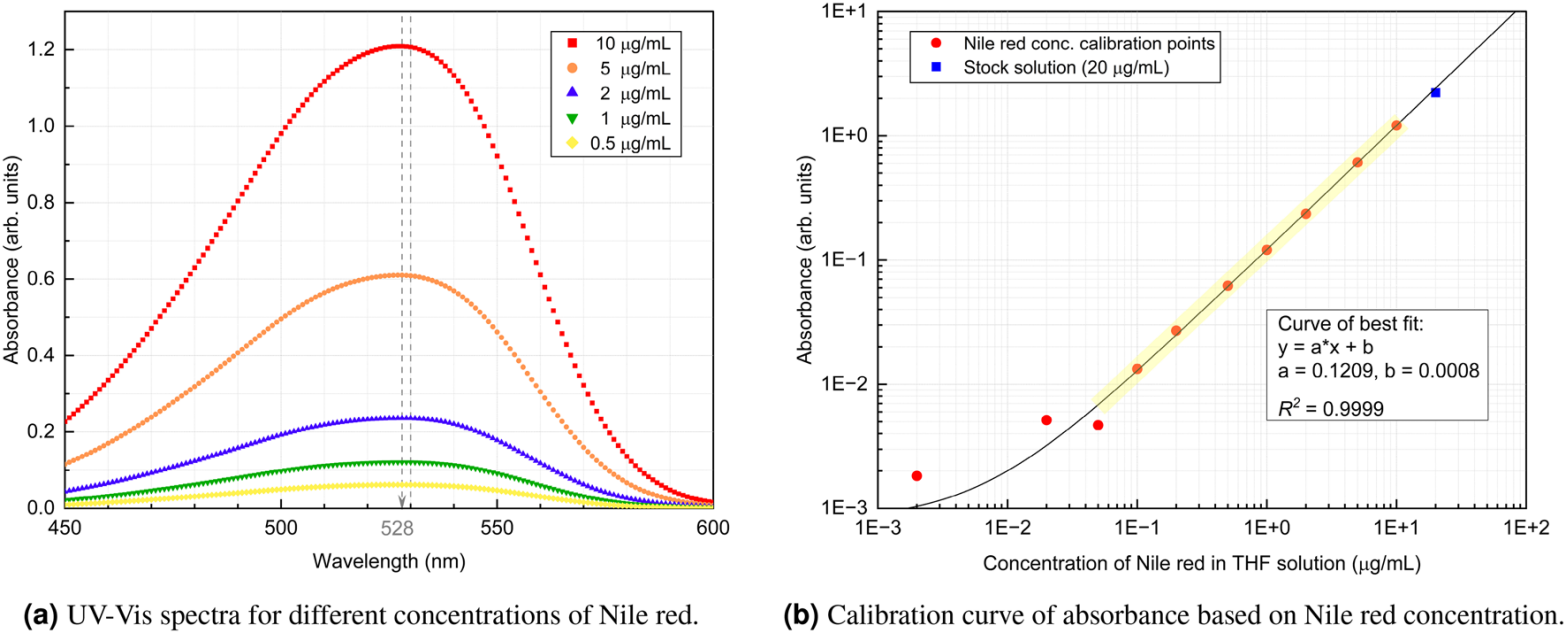
Absorbance spectrum (**a**), and final calibration curve (**b**) for Nile red solutions used to measure residual oil volume after displacement. The complete data set for UV–Vis spectra measurements (**a**), and the calibration curve (**b**) respectively can be found as Supplementary Tables S1 and S2 online.

A standard procedure to prepare solutions for the calibration curve was followed: The stock solution was first prepared with a concentration of 20 µg/mL Nile red in THF. Then a series of standard solutions were prepared by progressively diluting the stock solution with THF. This reduced the Nile red concentration to 10 µg/mL, 5 µg/mL, 2 µg/mL, 1 µg/mL, 0.5 µg/mL, 0.2 µg/mL, 0.1 µg/mL, 0.05 µg/mL, 0.02 µg/mL, and finally 0.002 µg/mL. The UV–Vis absorption spectrum of the solutions with 10 µg/mL, 5 µg/mL, 2 µg/mL, 1 µg/mL and 0.5 µg/mL Nile Red concentration are shown in Fig. 3a. The maximum absorbance peak needs to be identified to have the maximum sensitivity during measurements. As shown in Fig. 3a, the wavelength of maximum absorbance is found between 528 nm and 530 nm for all concentrations. To generate the calibration curve, the absorption of a given solution is calculated from the average of the absorption of the wavelength between 528 nm and 530 nm.

The absorbance of solutions with different concentration Nile red is shown in Fig. 3b. A least squares linear regression is performed to obtain the calibration curve shown in the figure. Denoting the absorbance by *y* (in arbitrary units) and the Nile red concentration (in units of µg/mL) by *x*, we find the calibration curve to be $y = 0.1029 x + 0.0008$ with a correlation coefficient of 0.999. This suggests that the linear relationship between absorbance and concentrations is excellent, as is observed on the graph. This calibration curve is used as a reference to calculate the concentration of Nile red in an unknown sample based on its measured absorbance. The calibration curve in Fig. 3b was also used to optimize the concentration of Nile red in the oil phase: To have effective experimental operation, a suitable concentration of Nile red needs to be selected so that the final solution, recovered at the end of the experiment, will have measurable absorbance and not be oversaturated. The optimal range of operation is shaded in Fig. 3b. With this in mind, we aimed at obtaining a concentration of Nile red of approximately 0.8 µg/mL in the final residual wall layer. Assuming that an equivalent uniform oil film thickness of 60 µm would be representative for several of our experiments, we added 28 mg Nile red to 175 mL mineral oil to arrive at this approximate level of dye in the residual oil at the end of the experiment.

As described above, the retrievable test section with the residual oil wall film was submerged in a fixed volume of THF to dissolve the oil and Nile red. Denoting this volume of solvent by $V_{THF}$, we estimate the residual oil volume, $V_{oil}$, as: $V_{oil} = C_{THF,NR} \cdot V_{THF} / C_{init,NR}$, where now $C_{init,NR}$ and $C_{THF,NR}$ denote the initial (known) concentration Nile red added to the mineral oil, and $C_{THF,NR}$ is the concentration Nile red in the recovered THF solution. Finally, we may convert the residual oil volume to an equivalent oil film thickness, *t*, by assuming a uniform wall film thickness along the length of the sample section, *i.e.* $V_{oil} = \pi \Delta L (a^2-(a-t)^2))$, with *a* the inner radius of the sample section and $\Delta L$ its length.

### Fluids and experiment conditions

We utilize a low-viscosity mineral oil, Sipdrill 4/0, provided by Halliburton, as the fluid to be displaced. As explained above, oil was stained by a known concentration of Nile red before displacement experiments were performed. Tap water was used as the displacing fluid, and relevant fluid properties are provided in Table 1.

**Table 1:** Fluid properties at $20\,^{\circ }$C.

Fluid	Density (kg/m$^3$)	Viscosity (mPa s)	Interfacial tension (mN/m)
Sipdril 4/0 (displaced fluid)	820	2.95	44.7
Water (displacing fluid)	998	1.00	

Displacement experiments using these fluids have been performed at two different imposed flow rates, and by injecting different volumes of displacing fluid. This allowed us to probe the possible effect of the flow rate on the residual oil volume, and to observe how the residual oil volume decreases as more displacing fluid is injected. We have performed experiments where the injected volume of displacing fluid varied from approximately 0.36 L up to 3.2 L. The total pipe volume is 0.18 L, so the displacing fluid volume ranged from 2 up to approximately 18 units of pipe volume.

For the current set of experiments, constant imposed flow rates of either 1.05 L/min and 1.45 L/min were used, corresponding to imposed bulk velocities of about 9.8 cm/s and 13.6 cm/s, respectively. In Table 2, we categorize experiments into three cases. The first two cases were performed at the same imposed flow rate, but with different initial concentration of Nile red in the oil phase. The experiments in these two campaigns were expected to produce the same residual oil volumes, and were motivated in part to validate the overall experimental methodology. The experiments in the third case were performed at a reduced imposed flow rate, and an initial Nile red concentration identical to case 1, and allowed us to observe whether the residual oil volume is sensitive to imposed flow rate. All experiments were repeated at least once to test for repeatability. Finally, we point out that the actual injected volumes reported below have been calculated from the flow meter measurement in each experiment. This is considered to provide the most precise estimate of the total injected volume.

**Table 2:** Categorization of experiments.

Case #	Concentration of nile red in oil (µg/mL)	Flow rate (L/min)
1	140	1.45
2	325	1.45
3	140	1.05

## Model for stratified pipe displacements

To facilitate interpretation of the experimental observations, we compare measurements of residual oil volume in the sampling section to predictions by a reduced-order displacement model. To motivate this model, we first assess the magnitude of stresses associated with inertia, buoyancy, fluid friction and interfacial tension, using the fluid properties and experiment conditions presented in Tables 1 and 2. We take $\tau ^* = {\bar{\rho }} U^{*2}$ as a typical inertial stress scale, with ${\bar{\rho }}$ the mean of the two fluid densities, and $U^*$ as the imposed bulk velocity. We define this as the ratio of imposed volumetric flow rate, *Q*, and the pipe cross sectional area, $A = \pi D^2 / 4$, *i.e.* $U^* = Q / A$. Depending on flow rate used, we estimate $\tau ^* \approx$ 8-17 Pa. The buoyant stress resulting from the density difference may be estimated as $\Delta \rho g D \approx 26$ Pa, with $\Delta \rho$ being the density difference between displacing and displaced fluid, and *D* being the pipe inner diameter.

**Figure 4:**
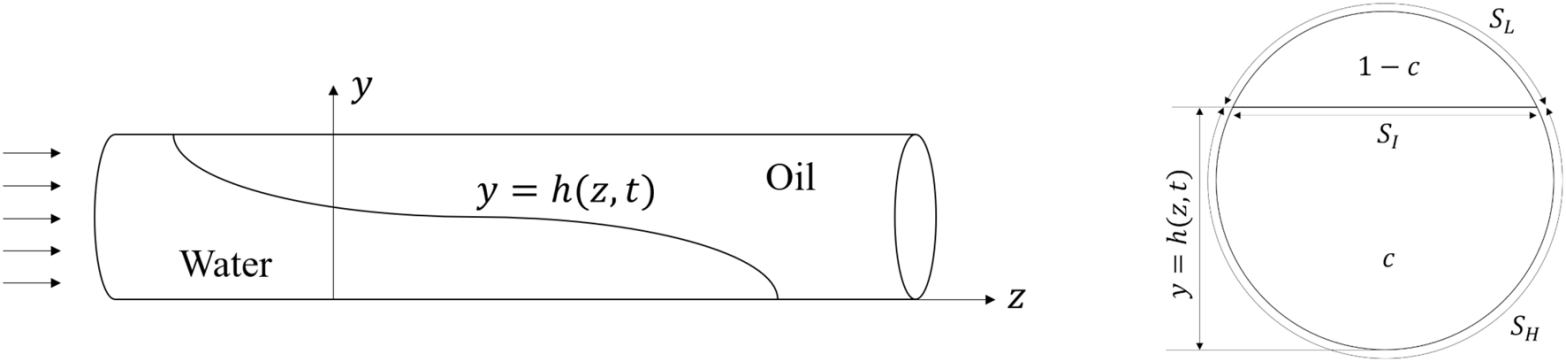
Schematic showing the assumed fluid configuration during displacement, where the density difference between the fluids results in a stratified interface, defined by a single-valued function $y = h(z,t)$, where *y* is in the vertical direction and *z* is parallel to the pipe axis. In a cross-sectional plane through the pipe, water occupies a fraction *c* of the cross-section, and wets a perimeter $S_H$ of the pipe. The lighter oil occupies the upper $1-c$ fraction of the cross-section and wets a perimeter $S_L$. The interface between the fluids is assumed horizontal within the cross-sectional plane, and of length $S_I$.

We next quantify viscous stresses using the geometric mean of the viscosities, ${\bar{\mu }} = \sqrt{\mu _H \mu _L}$, and obtain $\tau ^*_v = {\bar{\mu }} U^* / D \approx 0.01$ Pa for this stress scale. Finally, interfacial tension yields $\sigma / D \approx 3$ Pa. The considerable buoyant component promotes advancement of water along the bottom side of the pipe and with oil occupying the top side, as shown schematically in Fig. 4. The late time behavior of the displacement experiments was therefore likely in the form of a gradual draining of the top oil channel, where interfacial instabilities may accelerate the oil removal.

The assumed stratified fluid configuration shown in Fig. 4 motivates a classical long-wavelength lubrication scaling^20,21^, in which axial (streamwise) variations take place over a significantly longer length scale, $L^*$, compared to the pipe diameter, *D*, *i.e.* $\epsilon = D / L^* \ll 1$. Two-layer (stratified) models for buoyant pipe displacements within the lubrication approximation have been developed by *e.g.* Taghavi et al.^22^ and by Etrati and Frigaard^23^. We will in the following apply a simplified version of the latter model where we specialize to horizontal pipes and neglect inertial terms in the momentum equation for the two fluids. Before proceeding, we acknowledge that neglecting inertial and interfacial tension effects, as well as wall wetting effects, represents a considerable simplification of the actual experimental conditions.

Denoting by $Re = \tau ^* / \tau _v^*$ the Reynolds number of the flow, and neglecting terms of order $\epsilon$, $\epsilon Re$ and higher order in the governing equations, we have that the governing equations for the stratified displacements are (i) the continuity equation: $\mathbf {\nabla } \cdot \textbf{u} = 0$; (ii) momentum conservation for fluid *k*: $0 = -\mathbf {\nabla } p + \mathbf {\nabla } \cdot \varvec{\tau }_k + \rho _k \textbf{g}$; and (iii) the kinematic equation for the interface height *h* as measured from the bottom of the pipe: $D [h(z,t) - y] / Dt = 0$. As shown in the right panel of Fig. 4, the fluid interface is assumed flat in the pipe cross-section. We denote by $\textbf{u}$ the velocity vector, *p* the pressure, $\rho _k$ and $\varvec{\tau }_k$ are the mass density and deviatoric stress tensor of fluid *k*, and $\textbf{g}$ is the gravitational acceleration. In the following, we denote by *c*(*z*, *t*) the concentration of displacing fluid at axial position *z* and time *t*; equivalent to the area fraction occupied by the displacing fluid, as seen in Fig. 4.

Averaging the governing equations over the regions occupied by the displacing and displaced fluids, in accordance with the derivation of Etrati and Frigaard^23^, one arrives at a set of three equations for the unknown displacing fluid concentration, *c*(*z*, *t*), and the mean axial velocities $u_{H}$ and $u_{L}$ for the displacing (*H*) and displaced (*L*) fluid phases, respectively:

1 $$\begin{aligned} \partial _t c + \partial _z (u_{H} c)&= 0 \end{aligned}$$

2 $$\begin{aligned} c u_{H} + (1 - c) u_{L}&= U^* \end{aligned}$$

3 $$\begin{aligned} \frac{1}{A} \left[ \frac{\tau _H S_H + \tau _{iH} S_I}{c} + \frac{\tau _{iL} S_I - \tau _{L} S_L}{1 - c} \right]&= - \Delta \rho g \partial _z h. \end{aligned}$$

Here, $\tau _H$ and $\tau _L$ denote the wall shear stress for the displacing (*H*) and displaced fluid (*L*), respectively, and $\tau _{iH} (\tau _{iL})$ denotes the interfacial shear stress at the displacing fluid (displaced fluid) side of the interface^23^. As indicated in Fig. 4, $S_H$ and $S_L$ correspond to the pipe wall perimeter wetted by the displacing and the displaced fluid, respectively, and $S_I$ is the length of the horizontal interface in the cross-sectional plane of the pipe. By invoking suitable closure relations for these shear stresses, as listed in the “Appendix”, Eqs. (1), (2) and (3) can be combined to solve for the spatiotemporal evolution of *c*.

**Figure 5:**
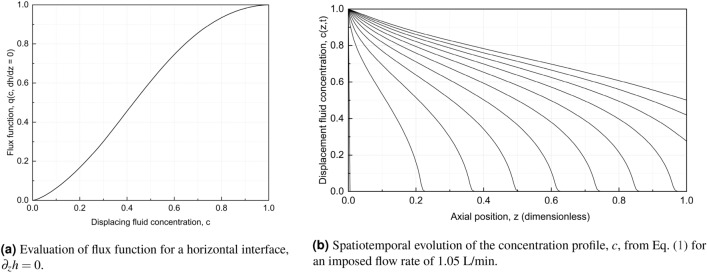
The spatiotemporal evolution of *c* is governed according to Eq. 1 by the flux function $u_H c$. In (**a**) we plot the flux function normalized by the imposed velocity, $q(c, \partial _z h) = u_H c / U^*$, in the limit of a perfectly horizontal interface, $\partial _z h = 0$. In (**b**), solutions of Eq. 1 for imposed flow rate corresponding to 1.05 L/min are shown as function of axial position normalized by the length of the pipe. The temporal separation of successive curves is $\Delta t = 0.08 L / U^*$.

In Eq. (1), $u_H c$ denotes the flux of displacing fluid, and we evaluate this flux for a fully stratified, horizontal interface ($\partial _z h = 0$) in Fig. 5a. We have here used the viscosity values for the two test fluids as tabulated in Table 1, and also normalized the flux by the imposed bulk velocity. Although the convex shape of the flux function in Fig. 5a may suggest shocks (discontinuities) in the solution for *c*, the density difference between the fluids (captured by the right-hand side of Eq. (3)) will act to spread the concentration profile in regions where $\partial _z h \ne 0$. A similar diffusive spreading due to gravity has been reported by *e.g.* Carrasco-Teja *et al.*^24^ within the context of horizontal annular displacements. In Fig. 5b, we present equitemporal concentration profiles plotted as function of axial position along the pipe normalized by the pipe length for an imposed flow rate of 1.05 L/min. The spatiotemporal concentration profiles are obtained by integrating Eq. (1) using a Lax-Friedrichs method^25^. The spatiotemporal evolution of the concentration profile shows expected behavior, i.e. an advancing front along the bottom of the pipe and where diffusive spreading due to buoyancy prevents the formation of a shock front, and a slow draining of the lighter oil from the top of the pipe. Slightly steeper but similar profiles are obtained for the higher flow rate of 1.45 L/min. Finally, to compare the model predicted residual oil volume after injecting a specific volume of displacing fluid, we average *c* over the length of the sampling section, corresponding to the interval [0.8, 0.87] in Fig. 5b.

## Results

**Figure 6:**
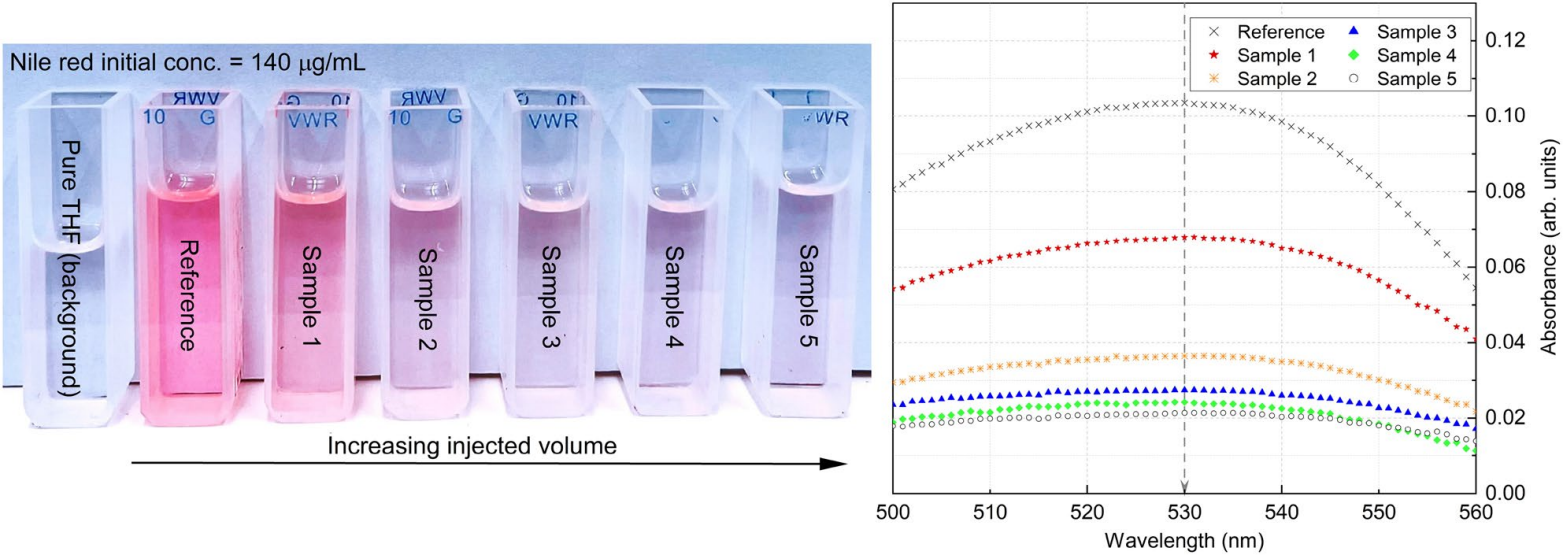
Pictogram of final THF solution in quartz cuvettes with different injected volume from Case #1 (left) and their UV–Vis spectra (right). The complete data set for UV–Vis spectra measurements (right) can be found as Supplementary Table S3 online.

Recovered THF solutions from experiments with different volumes of injected, displacing fluid are shown in the left panel of Fig. 6 (samples 1 through 5), along with a sample of pure THF and a reference sample. The reference sample was recovered by simply filling the pipe with Nile red stained mineral oil, and then draining the pipe before submerging the retrievable test section in THF solution. As such, the reference solution correspond to a case with no injected, displacing fluid, and instead represents the residual oil wall film following draining of oil. All samples shown in the figure were recovered from experiments belonging to Case #1 in Table 2, and the injected displacing fluid volumes increase from sample 1 to sample 5.

From the appearance of the recovered fluid samples, one can visually observe a dilution of the Nile red concentration from the reference sample and sample 1 to sample 5. As pointed out above, this corresponds to experiments where an increasing volume of displacing fluid has been injected, and the dilution corresponds to a smaller, recovered oil volume toward sample 5. While not shown here, recovered fluid samples from Cases #2 and #3 exhibit the same trend. Shown in the right panel of Fig. 6, is the UV–visible spectroscopy measurements for these samples. The peak of the measurements are located at a wavelength of approximately 530 nm, as expected based on the calibration curves shown in Fig. 3a. Further, the measured peak values decrease from the reference sample and sample 1 toward the lowest peak value, associated with sample 5.

**Figure 7:**
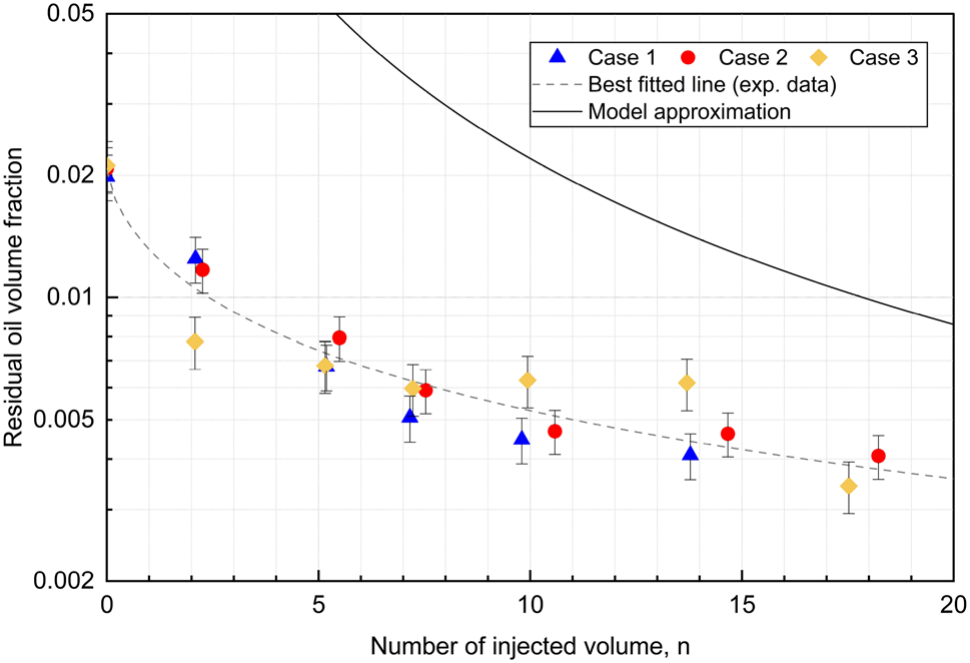
Measureded residual oil volume fraction as a function of injected pipe volumes, *n*. One pipe volume corresponds to approximately 0.18 L. The oil volume fraction and converted equivalent oil film thickness can be found as Supplementary Table S4 online. The model approximation curve, corresponding to an imposed flow rate of 1.05 L/min, is available as Supplementary Table S5 online.

We next infer the residual oil volume in the recovered fluid samples by comparing the UV–visible spectroscopy measurement to the calibration curve in Fig. 3b, and by correcting for the additional dilution due to the THF solvent. The measured residual oil volume is shown in Fig. 7 as function of the volume of injected, displacing fluid, *n*. All measurements from the three cases identified in Table 2 are included in this figure.

Measurements of the reference sample suggest a residual oil volume fraction of 0.02, which is corresponding to a wall film thickness of approximately 80 micrometer in all cases. Further, we observe for Cases #2 and #3 that the residual oil volume fraction as function of injected volume are showing a similar quantitative trend. Since it is only the initial Nile red concentration in the oil phase that varies between these two cases, this observation suggests that the measurement of residual oil volume is not dependent on the initial Nile red concentration, as expected. Next, experiments belonging to Case #3 were performed with a Nile red concentration equal to Case #1, but at a reduced flow rate of 1.05 L/min. Although results from experiments in Case #3 show some more variation compared to the other two cases, the general trend is considered to be the same, i.e. a progressive reduction in the residual oil volume with increasing injected volume of displacing fluid.

To estimate how the measured residual oil volume fraction depends on *n*, we pool all measurements and fit them to the empirical equation ${ V_{oil}}(n) = p/(1+qn^r)$. Here *p*, *q*, and *r* are model parameters, with *p* corresponding to the residual oil volume fraction for the reference sample and with $p(1+q)^{-1}$ being the residual oil volume fraction after injection of a volume $n = 1$ of displacing fluid. Finally, the empirical model suggests that ${ V_{oil}} \sim n^{-r}$ for large *n*. The dashed line in Fig. 7 correspond to the least squares estimates $p = 0.021$, $q = 0.57$ and $r = 0.71$. As such, the experimental measurements suggest a decrease of the residual oil volume that is slower than $n^{-1}$. The adjusted $R^2$ value is 0.96, suggesting a good fit. Further regression model details are provided in Table 3. The regression output shows that all three parameters *p*, *q*, and *r* are statistically significant.

**Table 3:** Summary of regression analysis results.

Formula: ${ V_{oil}} \sim p/(1 + qn^r)$				
Parameters	Estimates	Std. error	*t*-value	$Pr (> t)$)
*p*	0.0206	$6.2\cdot 10^{-5}$	31.109	$< 2\cdot 10^{-16}$
*q*	0.568	0.111	5.129	$8.37\cdot 10^{-5}$
*r*	0.709	0.095	7.451	$9.48\cdot 10^{-7}$

Finally, the solid curve included in Fig. 7 corresponds to the prediction by the reduced-order displacement model, defined by Eqs. (1), (2), (3) and the closure relations presented in the “Appendix”, for an imposed flow rate corresponding to 1.05 L/min. We observe from Fig. 7 that the model is conservative and over-predicts the residual oil volume within the sampling section. Further, the model predicts a $V_{oil} \sim n^{-1.4}$ for large *n*, which corresponds to a quicker volume reduction than that measured in experiments.

## Discussion

As noted above, and as expected, both experiments and the reduced-order model predicts a decrease in the residual oil volume as the total volume of injected, displacing fluid increases. The observed difference between prediction and measurement suggests that key displacement mechanisms are not included in the model formulation. A potentially significant effect is that of wettability of the inner pipe wall; carbon steel may show either metastable hydrophobicity or extreme hydrophilicity^26^, which may impact the displacement efficiency in experiments. A previous experimental study that investigated the immiscible displacement of a brine by oil in acrylic pipes, found pinning of the oil-brine interface at pipe wall, which resulted in a significant reduction in displacement efficiency^11^. Oladosu et al. suggest that a the displacement efficiency is very likely to improve (as we observe), when a wetting fluid displaces a non-wetting fluid^11^. A more systematic investigation of wetting properties of relevant pipe materials and their impact on residual oil volume will be targeted in future studies. From a modelling perspective, wall slip relations^27^ may improve the agreement between predicted and observed residual oil volumes. We also note that the reduced-order model does not include inertial terms in the momentum equation, which may be responsible for interface instabilities in our experiments. Future experiments will probe a wider range of flow rates and fluid viscosities in order to study possible inertial effects on the residual oil volume.

Although experiments show good repeatability and consistent trends, we note some sources of potential inaccuracies and measurement errors. Over the course of the experiments, we observed challenges in preparing a fully homogeneous and well-mixed stock solution of mineral oil and Nile red. That is, as the concentration of Nile red increases, it becomes harder to fully mix the Nile red into oil, and also more difficult to keep the oil well-mixed. Consequently, the oil that contains a higher concentration is more likely to have Nile red particles precipitate. These particles can get stuck on the walls of the sample section of the setup, which can result in a higher level of dye presented in the final solution. A related challenge is the sensitivity of the UV–Vis spectrophotometer to particularly small quantities of Nile red. This is reflected as slightly larger error bars for the larger values of *n* in Fig. 7. It is a well-known limitation of UV-spectrophotometry that its sensitivity is often inadequate for very low sample concentrations. In this case, when the volume of injected displacing fluid increases, the wall layer thickness decreases, which results in a lower level of Nile red concentration in the final solution. This in turn, enlarges the uncertainty of measurements. Another unavoidable source of error comes from experimental operation, when at the end of each experiment, the pipe will be drained prior to detaching the sampling section. This draining of the pipe can potentially cause a loss of residual oil.

Finally, we have identified several steps need to be addressed or monitored to ensure accurate and repeatable results in future experimental campaigns:

- Mixing of the oil: The homogeneity of dyed oil is critical for this methodology to work. In this work, all the oil mixing is done by an ultrasonic homogenizer. This method, however, only works for a small sample of oil. When requiring a large volume of oil, ultrasonic homogenizing will not be an effective mixing method. An alternative that could work for upscaling this methodology is to first dissolve the Nile red dye in a strong organic solvent (such as THF), and then mix this solution into the oil sample. Since Nile red will easily dissolve in the organic solvent, this should enable a practical method for adding the dye to the oil phase. The last step is to remove the THF solvent by vaporization.
- Possible contamination during experimental operation: Due to the miniscule volume of residual oil left after displacement, the measured wall layer thickness will be very sensitive to mass of Nile red in the THF solution. During operation, it is therefore very important to make sure that there is no oil contamination on the surface of any parts involved. This can be achieved by scrubbing the sampling section with acetone solution before and after each run of the experiment.
- Initial oil concentration: Measurement of the UV–vis absorption spectrum is less sensitive to the Nile red concentration when its concentration is low. Thus, deciding on a suitable initial Nile red concentration in the oil-phase is important in order to obtain accurate results. In our case, the ideal initial concentration to run future experiments should be around 140 µg/mL. From the calibration curve, this initial concentration will guarantee a good absorption reading from UV–vis measurements, and at the same time will limit the sources of contamination during experiments.

## Conclusion

In this paper, we have presented a method for quantifying the residual oil volume in a pipe following displacement by a known volume of an immiscible, displacing fluid. The method relies on using a hydrophobic dye to stain the oil phase, and to use UV–visible spectrometry to measure the residual oil volume after displacement. This method effectively addresses the long-standing challenge of quantitatively measuring micrometer level oil films thickness with a simple and fast experimental instrumentation. To validate the proposed methodology, three sets of displacement experiments were performed. For each set of experiments, the concentration of Nile red in oil and the flow rate of displacing fluid were fixed, while the injected volume was progressively increased. In theory, the variation of concentration should not have any effects on the final calculated wall layer thickness. It was also anticipated from scaling arguments that the variation of laminar flow rate of displacing fluid should have a small effect on the final wall layer thickness, and that the film thickness is more sensitive to the injected volume of displacing fluid. The experimental results confirm this theoretical hypothesis. These results validate the feasibility and repeatability of the experimental method. When compared to the predictions of a stratified and non-inertial displacement model, the experimental data produces consistently smaller residual oil volumes. We believe this may be ascribed to inertial and wetting effects which are not accounted for in the model. Future work will investigate these effects in greater detail, in addition to studying how displacing fluid viscosity, different surface materials and a larger range of imposed flow rates (laminar, transitional and turbulent regimes) impact on the residual oil volume.

### Supplementary Information

Supplementary Information 1.

Supplementary Information 2.

Supplementary Information 3.

Supplementary Information 4.

Supplementary Information 5.

## Data Availability

Additional datasets generated during and/or analysed during the current study are available from the corresponding author on reasonable request.

## Appendix

### Frictional closure relations

Closure relations for the wall and interface stresses in Eq. (3) are needed in order to advance the concentration profile according to Eq. (1). In this study, we have adopted Model 2 in the two-layer study of Etrati and Frigaard^23^ where the dimensional wall stresses of the displacing (*H*) and displaced (*L*) read:

$$\begin{aligned} \tau _H&= \frac{8 \mu _H u_H}{D} \frac{S_H + S_I}{c \pi D} F_H, \qquad F_H = \frac{1}{1 + X} \left( 1 + g_{11}X - g_{12}u_L/u_H \right) , \\ \tau _L&= \frac{8 \mu _L u_L}{D} \frac{S_L + S_I}{(1 - c) \pi D} F_L, \qquad F_L = \frac{X}{1 + X} \left( 1 + g_{22}/X - g_{21} u_H/u_L\right) . \end{aligned}$$

Here, $$X = \mu _H (1-c) / (\mu _L c)$$, and

$$\begin{aligned} g_{11} = \frac{S_H}{S_H + S_I}, \qquad g_{12} = \frac{4}{2 + \pi } \frac{S_H}{S_H + S_L}, \qquad g_{21} = \frac{4}{2 + \pi } \frac{S_L}{S_H + S_L}, \qquad g_{22} = \frac{S_L}{S_L + S_I}. \end{aligned}$$

Finally, the interfacial shear stress is taken as^23^

$$\begin{aligned} \tau _i = \left\{ \begin{array}{l l} \frac{8 \mu _H (u_H - u_L)}{D} \frac{S_H + S_I}{c\pi D} \frac{1}{1 + X} &{} \text { if } u_H > u_L, \\ \frac{8 \mu _L (u_H - u_L)}{D} \frac{S_L + S_I}{(1 - c) \pi D} \frac{1}{1 + 1/X} &{} \text { if } u_H < u_L. \end{array} \right. \end{aligned}$$
